# lncSAMM50 Enhances Adipogenic Differentiation of Buffalo Adipocytes With No Effect on Its Host Gene

**DOI:** 10.3389/fgene.2021.626158

**Published:** 2021-03-26

**Authors:** Ruirui Zhu, Xue Feng, Yutong Wei, Duo Guo, Jiaojiao Li, Qingyou Liu, Jianrong Jiang, Deshun Shi, Jieping Huang

**Affiliations:** ^1^State Key Laboratory for Conservation and Utilization of Subtropical Agro-Bioresources, Guangxi University, Nanning, China; ^2^College of Life Sciences, Xinyang Normal University, Xinyang, China

**Keywords:** *Bubalus bubalis*, adipose, RNA sequencing, long non-coding RNA, adipogenesis

## Abstract

Fat deposition is one of the most important traits that are mediated by a set of complex regulatory factors in meat animals. Several researches have revealed the significant role of long non-coding RNAs (lncRNAs) in fat deposition while the precise regulatory mechanism is still largely elusive. In this study, we investigated the lncRNA profiles of adipose and muscle tissues in buffalo by using the Illumina HiSeq 3000 platform. In total, 43,809 lncRNAs were finally identified based on the computer algorithm. A comparison analysis revealed 241 lncRNAs that are differentially expressed (DE) in adipose and muscle tissues. We focused on lncSAMM50, a DE lncRNA that has a high expression in adipose tissue. Sequence alignment showed that lncSAMM50 is transcribed from the antisense strand of the upstream region of sorting and assembly machinery component 50 homolog (*SAMM50*), a gene involved in the function of mitochondrion and is subsequently demonstrated to inhibit the adipogenic differentiation of 3T3-L1 adipocyte cells in this study. lncSAMM50 is highly expressed in adipose tissue and upregulated in the mature adipocytes and mainly exists in the nucleus. Gain-of-function experiments demonstrated that lncSAMM50 promotes the adipogenic differentiation by upregulating adipogenic markers but with no effect on its host gene *SAMM50* in buffalo adipocytes. These results indicate that lncSAMM50 enhances fat deposition in buffalo and provide a new factor for the regulatory network of adipogenesis.

## Introduction

The buffalo (*Bubalus bubalis*) is a globally important domestic animal providing economic value from meat, milk, and draft power. In China, the number of buffaloes is more than 27 million, second only to India and Pakistan (FAO, http://www.fao.org/, 2019). Traditionally, buffaloes are raised for draught power in China. Recently, with the increasing agricultural mechanization, the utility of buffaloes in draught power has gradually decreased, indicating that the role of buffaloes can be changed into a meat source (Kiran and Naveena, 2014). The fat deposition level in Chinese buffalo is very low due to the long-term breeding for draught power. However, both backfat thickness and intramuscular fat (IMF) content, which are associated with fat deposition, are vital traits for meat animals as buffalo. Especially, IMF content is highly correlated with tenderness, juiciness, and flavor of buffalo meat. A lower backfat thickness and a higher IMF content are of benefit to beef production. However, it is nearly impossible to decrease the backfat thickness and to increase the IMF deposition at the same time, indicating that the regulatory mechanism of fat deposition is far from complete to be understood, as new regulatory factors need to be discovered.

In animals, excess energy is stored as triglycerides within the lipid droplets of adipocytes and then expressed as fat deposition. Adipogenesis is the process of cell differentiation from preadipocytes to mature adipocytes, with lipid accumulation in cells. This process has been widely studied for decades. Researches *in vitro* and *in vivo* show that adipogenesis is a highly complex process that can be regulated by a large number of factors (Lowe et al., 2011; Mota de Sá et al., 2017). Peroxisome proliferator-activated receptor gamma (PPARγ or PPARG) is the most well-studied one and is undoubtedly the most significant modulator in adipogenesis of animals (Lowe et al., 2011; Mota de Sá et al., 2017). Many other factors, such as the CCAAT/enhancer-binding protein family (C/EBPs; Cao et al., 1991; Yeh et al., 1995; Hamm et al., 2001), Kruppel-like transcription factors (KLFs; Mori et al., 2005; Oishi et al., 2005; Birsoy et al., 2008), and GATA transcription factors (Tong et al., 2000, 2005; Jack and Crossley, 2010), have also been identified as important modulators in adipogenesis. However, most evidences are based on studies in humans and model animals as rodents. In buffaloes, researches on adipogenesis are still very limited. The genetic diversities of adipogenesis relative genes have been suggested to be with the adipogenesis of milk fat (Gu et al., 2017, 2019, 2020). Phosphoenolpyruvate carboxykinase 1 has been identified as a significant candidate gene that is involved in IMF deposition by transcriptome sequencing analysis and functional validation in buffalo adipocytes (Huang et al., 2020).

Although the major regulatory activity of adipogenesis has been revealed, the precisely orchestrated process is far from complete, as new modulators in this process are gradually identified. In recent years, increasing long non-coding RNAs (lncRNAs) have been demonstrated to have profound effects on adipogenesis (Li et al., 2016; Huang et al., 2019). lncRNAs are a kind of well-known non-coding RNAs that have more than 200 nucleotides and have become a research hotspot in recent years. With the development of high-throughput sequencing technology, increasing lncRNAs have been demonstrated to modulate fat deposition (Nuermaimaiti et al., 2018; Huang et al., 2019; Zhang and Fu, 2020; Zhang S. et al., 2020). The majority of studies that reveal a significant role of lncRNAs in adipogenesis are performed in humans (Nuermaimaiti et al., 2018; Zhang T. et al., 2020) or murine (Cai et al., 2018; Chen et al., 2020). In livestock animals, several lncRNAs also have been identified to modulate adipogenesis. In pigs, knockdown lncIMF4 promotes adipogenesis by attenuating autophagy to repress the lipolysis in intramuscular adipocytes (Sun et al., 2020). In cattle, lncRNA ADNCR suppresses adipogenic differentiation by targeting miR-204 (Li et al., 2016). Recently, a new lncRNA lncFAM200B is found to have an important role in the development of adipocytes in cattle (Zhang S. et al., 2020). In buffaloes, the *NDUFC2*-AS lncRNA promotes adipogenic differentiation by upregulating adipogenesis relative genes (Huang et al., 2019). Compared to the larger number of lncRNAs identified in adipose tissue (Huang et al., 2019), the number of present identified lncRNAs with effects on adipogenesis is very limited, suggesting that the modulatory role of lncRNAs is still poorly understood.

To uncover novel lncRNAs involved in the regulatory network of adipogenesis, lncRNA profiles of adipose and muscle tissues were characterized by high-throughput RNA sequencing using the Illumina HiSeq 3000 platform in this study. Differential expression analysis was performed, and the host gene was revealed to yield candidate lncRNAs with putative effects on adipogenesis. Further gain-of-function experiments demonstrated that an lncRNA, which transcribed from the upstream region of sorting and assembly machinery component 50 homolog (*SAMM50*), promotes the adipogenic differentiation of buffalo adipocytes by upregulating the adipogenesis relative gene. This study further supplies the buffalo lncRNA data and proposes a novel lncRNA that has a significant role in fat deposition of buffalo.

## Materials and Methods

### Animals and Sample Preparation

Chinese swamp buffaloes (bull, *n* = 3) were raised under equivalent forage and feeding management condition in Xinyang Buffalo Breeding Farm (Guangshan, Henan province, China) as previously described (Huang et al., 2019). Animals were weaned at 6 months of age and slaughtered at 30 months of age. Tissues (the longissimus dorsi muscle, back subcutaneous fat, heart, liver, spleen, lung, and kidney) were sampled immediately after slaughter and were frozen in liquid nitrogen for RNA sequencing and qRT-PCR experiments. Meanwhile, the fresh back subcutaneous fat was kept at ∼30°C in phosphate-buffered saline (PBS) with 1% streptomycin and penicillin and taken back to the lab for primary adipocyte isolation.

### RNA Isolation and Sequencing

Total RNA was isolated by TRIzol (Invitrogen, Carlsbad, CA, United States) according to the manufacturer’s instructions. RNA quantity was measured with NanoDrop 2000 (NanoDrop, Wilmington, DE, United States) and 1.5% agarose gels. RNA with 1.8 < 260/280 value <2.0 and concentration >500 ng/μL was used for further analysis. Isolation of nuclear and cytoplasmic RNA was performed by PARIS kit (Life Technologies, Carlsbad, CA, United States) according to the manufacturer’s instructions. Details of RNA isolation and high-throughput RNA sequencing were described previously (Huang et al., 2019). The longissimus dorsi muscle (*n* = 3) and the back subcutaneous fat (*n* = 3) were used for RNA sequencing.

### Quality Control, Transcriptome Assembly, lncRNA Prediction, and Differential Expression Analysis

Quality control, transcriptome assembly, and lncRNA prediction were performed as previously described (Huang et al., 2019). Briefly, the low-quality reads and those containing adapters were removed to obtain clean reads. Then, clean reads that are high-quality were used for the subsequent analysis. The cattle genome (UMD3.1) was used as the reference, for the annotation information of buffalo genome is not available. Clean reads were mapped to the reference genome to obtain complete transcripts. Transcripts with more than 200 bp and without coding capability were identified as lncRNAs. The expression level of lncRNA was indicated as log2(FPKM+1). lncRNA with the absolute value of log2(fold change) ≥ 2 and the FDR value ≤ 0.05 was considered to be differentially expressed (DE).

### qRT-PCR Analysis

Details of primer design, reverse transcription reaction, and quantitative PCR were described in our previous study (Huang et al., 2019). The ubiquitously expressed prefoldin-like chaperone (*UXT*) gene and the glyceraldehyde-3-phosphate dehydrogenase (*GAPDH*) gene were used to normalize the expression level of the candidate gene in tissues and adipocytes of buffalo, respectively (Huang et al., 2019; Feng et al., 2020). For the 3T3-L1 cells, β*-actin* was used as the internal reference gene. The cycle threshold (2^–ΔΔCt^) method was used to calculate the relative expression level of the candidate gene. In particular, for cell localization, *U6* and β*-actin* were respectively used as nuclear and cytoplasmic markers, and the 2^–ΔCt^ method was used to calculate the gene expression level. Three replicates were run per sample, and the qRT-PCR experiment was performed three times. Details of the primers used for qRT-PCR are shown in Supplementary Table 1.

### Vector Construction

The CDS region of mouse *SAMM50* (NCBI Reference Sequence: NM_178614.5) was amplified from the cDNA of mouse muscle tissue, which was kindly provided by Dr. Yongjie Xu of Xinyang Normal University (Xinyang, China) and cloned into the *Hin*dIII and *Xho*I restriction sites of pcDNA3.1(+) vector. Primers used to amplify the CDS region were as follows: F-5′-CCC*aagctt*GCCGAGCCTCTTGTGTTTG-3′; R-5′-CCGctcgagCCAGAAGCACTCAACCGTGT-3′. The lowercase indicates the restriction enzyme site.

### Cell Culture

The 3T3-L1 preadipocytes were purchased from ATCC (Shanghai, China). Buffalo primary adipocytes were isolated from adipose tissues of male buffaloes (*n* = 3) using the tissue block method as described in our previous study (Huang et al., 2019). Buffaloes used here were different than those used for RNA sequencing, but all the animals were raised under equivalent forage and feeding management conditions in the same farm and slaughtered at similar months of age. Adipocytes were cultured with a complete culture medium [Dulbecco’s modified Eagle’s medium (DMEM) with 10% fetal bovine serum and 1% penicillin–streptomycin] in 5% CO_2_ at 37°C. All the reagents used for cell culture were purchased from Gibco (Grand Island, NY, United States). Before transfection and transduction, cells were plated in a 6-well plate in triplicate.

### Transfection, Adipogenic Differentiation, Oil Red O Staining, and Quantification

For the 3T3-L1 preadipocytes, transfection was conducted when the cells reached 80% confluence by using Lipofectamine 3000 (Invitrogen, Carlsbad, CA, United States) following the manufacturer’s protocol. Two days after transfection, cells were induced to adipogenic differentiation treatment with an inducing medium (containing 10 μg/mL insulin, 1 μM rosiglitazone, 1 μM dexamethasone, and 0.5 mM IBMX). Two days later, cells were treated with a maintenance medium which contains 10 μg/mL insulin and 1 μM rosiglitazone. Meanwhile, transfection was performed again. After inducing with adipogenic agents for 8 days, Oil Red O staining and quantification were performed as previous described (Huang et al., 2019).

### Adenovirus Packaging and Transduction

Adenovirus packaging was performed at Hanbio Biotechnology Co., Ltd. (Shanghai, China). Briefly, the full length of lncSAMM50 was synthesized and ligated to the AdMax system to obtain Ad-lncSAMM50. EGFP was used as an internal indicator. Ad-EGFP was used as a negative control.

Similar to transfection, adenoviral transduction was conducted when the buffalo adipocytes reached 80% confluence. Twenty-four hours later, cells were treated with an inducing medium for 2 days and then treated with a maintenance medium for 4 days. The maintenance medium was changed every 2 days. After inducing with adipogenic agents for 6 days, Oil Red O staining and quantification of lipid content were performed as previously described (Huang et al., 2019).

### Statistical Analysis

Comparison was analyzed by using the SPSS 19.0 software. Student’s *t*-test was used when the data had a normal distribution; otherwise, a non-parametric test was performed. A value of *p* < 0.05 was considered to indicate statistically significant differences. Data were presented as mean ± SD by using the OriginPro 8.5 program.

## Results

### Differential Expression Analysis and Validation

In total, 43,809 lncRNAs were identified by a computer algorithm in buffalo adipose and muscle tissues in this study (Supplementary Table 2). Differential expression analysis revealed that 241 lncRNAs were DE between adipose and muscle tissues in buffalo (Supplementary Table 3). Among them, 125 were upregulated in adipose tissue compared with muscle tissue while others were downregulated (Supplementary Table 3).

**FIGURE 1:**
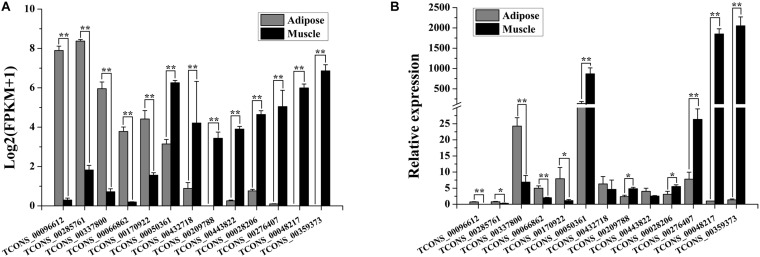
Validation of differentially expressed (DE) lncRNAs by qRT-PCR. **(A)** Expression patterns of the 13 DE lncRNAs in the adipose and muscle tissues of buffalo based on RNA sequencing. The expression level of lncRNA is indicated as log2(FPKM+1). Data are presented as mean ± SD (*n* = 3; *p < 0.05; **p < 0.01). **(B)** Expression patterns of the 13 DE lncRNAs in the adipose and muscle tissues of buffalo analyzed by qRT-PCR. The RNA expression levels are normalized to those of *UXT*. Data are presented as mean ± SD (*n* = 6; *p < 0.05; **p < 0.01).

To evaluate the quality of differential expression analysis, 13 lncRNAs (5 lncRNAs were upregulated and 8 were downregulated in adipose tissue) were randomly selected for validation by qRT-PCR. As shown in Figure 1, the expression patterns of 5/5 upregulated and 6/8 downregulated lncRNAs in qRT-PCR analysis were consistent with that in RNA sequencing analysis.

### Candidate lncRNAs Associated With Fat Deposition in Buffalo

The aim of this study was to identify lncRNAs with significant effect on fat deposition in buffalo. We noticed that four DE lncRNAs have log2(fold change) ≥ −5 and showed a high expression level in adipose tissue (Table 1). Among them, TCONS_00096612, TCONS_00285761, and TCONS_00337800 are transcribed from the antisense strand of fatty acid-binding protein 4 (*FABP4*), ubiquinone oxidoreductase subunit C2 (*NDUFC2*), and *SAMM50* gene, respectively. Interestingly, these genes have been confirmed to be associated with fat deposition. In addition, the *p* value for lncSAMM50 and *NDUFC2*-AS lncRNA was the lowest. Thus, we further focused on the effect of lncSAMM50 on the fat deposition in buffalo.

**TABLE 1:** Candidate lncRNAs associated with fat deposition in buffalo.

Transcript _id	Host gene	Strand	Symbol	Adipose _1	Adipose _2	Adipose _3	Muscle _1	Muscle _2	Muscle _3	Mean _adipose	Mean _muscle	Log2 (Fold Change)	*p*-value	FDR
TCONS _00096612	FABP4	Antisense strand	FABP4-AS lncRNA	7.49	8.25	7.94	0.12	0.47	0.30	7.89	0.29	−7.60	0.0034	0.0188
TCONS _00285761	NDUFC2	Antisense strand	NDUFC2-AS lncRNA	8.52	8.25	8.36	1.35	2.07	2.05	8.38	1.82	−6.56	0.0001	0.0004
TCONS _00285845	Intergenic region	–	–	6.99	7.19	6.29	0.78	0.90	1.54	6.82	1.07	−5.75	0.0014	0.0088
TCONS _00337800	SAMM50	Antisense strand	lncSAMM50	6.59	5.85	5.43	0.46	0.99	0.69	5.96	0.71	−5.24	0.0001	0.0004

### Characterization of lncSAMM50

**FIGURE 2:**
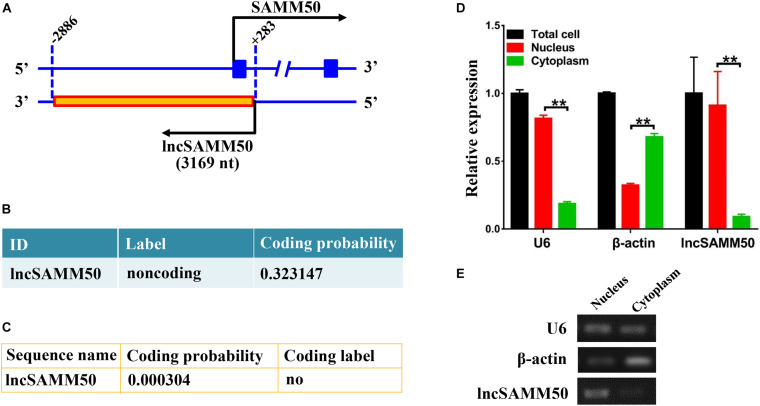
Characterization of buffalo lncSAMM50. **(A)** Positional relationship between SAMM50 and lncSAMM50. **(B)** The Coding Potential Calculator (CPC) program suggests that lncSAMM50 is a non-coding RNA. **(C)** The Coding Potential Assessment Tool (CPAT) indicates that lncSAMM50 is a non-coding RNA. **(D)** Cell localization of lncSAMM50 by qRT-PCR. Adipocytes induced to differentiation for 6 days were used for separation of nucleus and cytoplasm RNA. *U6* and β*-actin* were respectively used as nuclear and cytoplasmic markers. The 2^–ΔCt^ method was used to calculate the gene expression level. Data are presented as the mean ± SD (*n* = 3; **p < 0.01). **(E)** Cell localization of lncSAMM50 by semiquantitative PCR.

The full length of lncSAMM50 is 3,169 nt (Supplementary Table 4), and the sequence is reverse complementary with the upstream region, exon 1, and part of intron 1 of *SAMM50* (Figure 2A). Both Coding Potential Calculator (CPC) and Coding Potential Assessment Tool (CPAT) indicated that lncSAMM50 is a non-coding RNA (Figures 2B,C). Results of semiquantitative PCR for nuclear and cytoplasmic fractions showed that lncSAMM50 was mainly expressed in the nucleus (Figure 2E). The qRT-PCR detection confirmed that the expression pattern of lncSAMM50 was the same as a nuclear marker U6 (Figure 2D).

### Expression Pattern of lncSAMM50 and *SAMM50*

**FIGURE 3:**
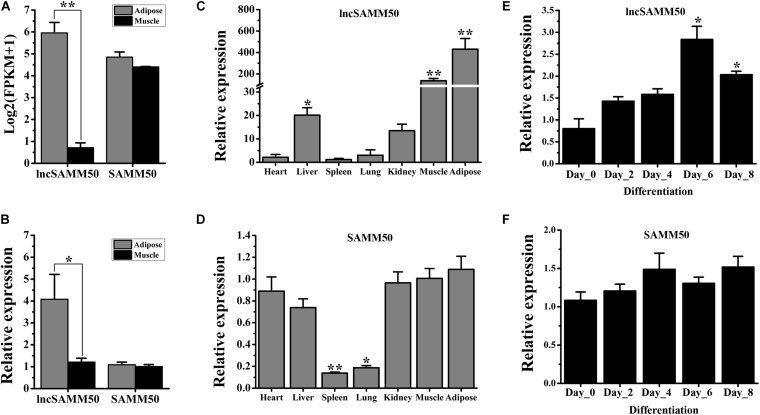
Expression pattern of lncSAMM50 and SAMM50 in buffalo. **(A)** The expression pattern of lncSAMM50 and *SAMM50* in adipose and muscle tissues by RNA sequencing. **(B)** The expression pattern of lncSAMM50 and *SAMM50* in adipose and muscle tissues by qRT-PCR. **(C)** The expression profile of lncSAMM50 in heart, liver, spleen, lung, kidney, muscle, and adipose tissues. (D) The expression profile of SAMM50 in heart, liver, spleen, lung, kidney, muscle, and adipose tissues. For panels **(A–D)**, Xinyang buffalo (30 months of age, *n* = 3) was used; the *UXT* gene was used to normalize the expression level of the candidate gene. **(E)** The expression pattern of lncSAMM50 during adipocyte differentiation. **(F)** The expression pattern of *SAMM50* during adipocyte differentiation. *GAPDH* was used to normalize the expression level of the candidate gene in adipocytes. The cycle threshold (2^–ΔΔCt^) method was used to calculate the relative expression level of the candidate gene. Data are presented as the mean ± SD (*n* = 3; *p < 0.05; **p < 0.01).

Based on RNA sequencing, the expression level of lncSAMM50 in adipose tissue is higher than that in muscle tissue (Figure 3A, *p* < 0.01), which was further conformed by qRT-PCR analysis (Figure 3B, *p* < 0.05). By contrast, *SAMM50*, the host gene of lncSAMM50, showed a similar expression level in adipose and muscle tissues (Figures 3A,B). Analysis of the tissue expression profile revealed that lncSAMM50 is mainly expressed in adipose and muscle tissues while *SAMM50* is widely expressed in variable tissues (Figures 3C,D). During adipogenic differentiation, lncSAMM50 was upregulated in the mature adipocytes of buffalo (Figure 3E) while *SAMM50* was widely expressed in different stages (Figure 3F).

### *SAMM50* Inhibits the Adipogenic Differentiation of 3T3-L1 Cells

**FIGURE 4:**
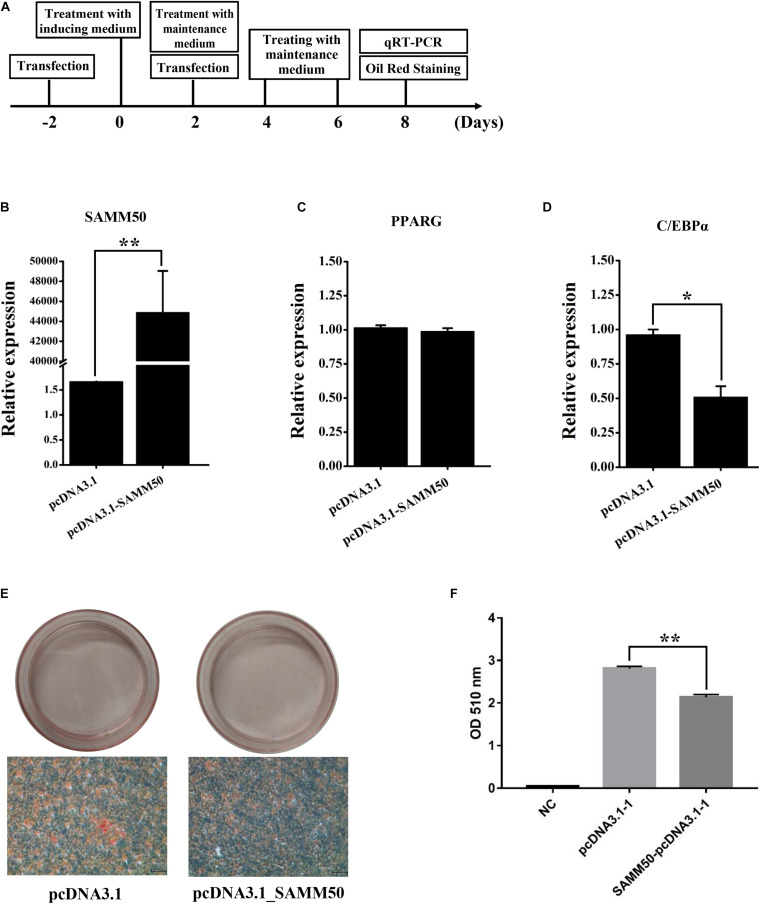
Overexpression of mouse SAMM50 inhibits adipogenic differentiation of 3T3-L1 adipocytes. **(A)** Strategy of *SAMM50* overexpression, adipogenic differentiation, and Oil Red O staining in 3T3-L1 adipocytes. **(B–D)** RNA expression of SAMM50, PPARG, and C/EBPα 48 h after transfection. *GAPDH* was used to normalize the expression level of the candidate gene in 3T3-L1 cells. The cycle threshold (2^–ΔΔCt^) method was used to calculate the relative expression level of the candidate gene. **(E)** Images of Oil Red O staining in 3T3-L1 cells transfected with pcDNA3.1 and pcDNA3.1-SAMM50 on day 8 of adipogenic differentiation. Scale bar, 200 μm. **(F)** Histogram showing the quantitation of Oil Red O staining by spectrophotometry. NC, negative control. Data are presented as the mean ± SD (*n* = 3; *p < 0.05; **p < 0.01).

To access the function of *SAMM50* in fat deposition, gain-of-function experiments for *SAMM50* were performed in 3T3-L1 adipocytes. The strategy of transfection, adipogenic differentiation, and Oil Red O staining is shown in Figure 4A. As expected, the mRNA expression of *SAMM50* was highly significantly upregulated in pcDNA3.1_SAMM50 group (Figure 4B, *p* < 0.01). Meanwhile, *C/EBP*α was significantly downregulated in the pcDNA3.1_SAMM50 group (Figure 4D, *p* < 0.05). Accordingly, lipid accumulation in the pcDNA3.1_SAMM50 group was less than that in the pcDNA3.1 group (Figures 4E,F). No effect was detected on the expression of *PPARG* (Figure 4C).

### lncSAMM50 Promotes the Adipogenic Differentiation of Buffalo Adipocytes

**FIGURE 5:**
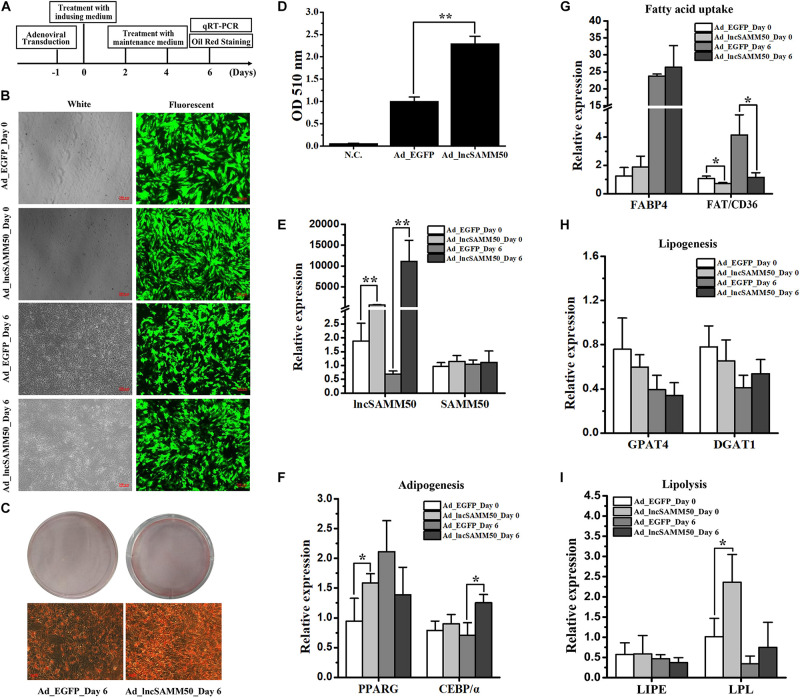
Overexpression of lncSAMM50 enhances adipogenic differentiation of buffalo adipocytes. **(A)** Strategy of lncSAMM50 overexpression, adipogenic differentiation, and Oil Red O staining in buffalo adipocytes. **(B)** Micrographs of EGFP-positive cells in the ad_EGFP (control) and ad_lncSAMM50 groups on day 0 and day 6 of adipogenic differentiation. Scale bar, 200 μm. **(C)** Images of Oil Red O staining in buffalo adipocytes transduced with ad_EGFP and ad_lncSAMM50 on day 6 of adipogenic differentiation. Scale bar, 50 μm. **(D)** Histogram showing the quantitation of Oil Red O staining by spectrophotometry. NC, negative control. **(E–I)** The RNA expression levels of lncSAMM50, SAMM50, PPARG, C/EBPα, FABP4, FAT/CD36, GPAT4, DGAT1, LIPE, and LPL on day 0 and day 6 of adipogenic differentiation in buffalo adipocytes transduced with ad_EGFP and ad_lncSAMM50. *GAPDH* was used to normalize the expression level of the candidate gene in buffalo adipocytes. The cycle threshold (2^–ΔΔCt^) method was used to calculate the relative expression level of the candidate gene. Data are presented as the mean ± SD (*n* = 3; *p < 0.05; **p < 0.01).

To evaluate the effect of lncSAMM50 on fat deposition in buffalo, the full length of lncSAMM50 (Supplementary Table 4) was packaged into an adenovirus system for overexpression (ad_lncSAMM50). The time axis of overexpression of LncSAMM50, induction, quantification is shown in Figure 5A. Indicator EGFP was highly expressed 1 day after adenoviral transduction and continued until the 6th day of adipogenic induction (Figure 5B). The expression of lncSAMM50 in the ad_lncSAMM50 group was significantly higher than that in the ad_EGFP group, and the overexpression was continued until the 6th day of adipogenic induction (Figure 5E, *p* < 0.01). Meanwhile, lipid accumulation in the ad_lncSAMM50 group was significantly enhanced (Figures 5C,D, *p* < 0.01). As to the adipogenic markers, the mRNA expressions of *PPARG* and *C/EBP*α were slightly upregulated on day_0 and day_6 of adipogenic induction, respectively (Figure 5F). Lipoprotein lipase (*LPL*), a lipolysis gene, was upregulated on day_0 of adipogenesis induction (24 h after lncSAMM50 overexpression) in the ad_lncSAMM50 group (Figure 5I). Confusingly, the fatty acid transporter (*FAT/CD36*), a fatty acid uptake marker, was downregulated in the ad_lncSAMM50 group (Figure 5G). For the expression of the host gene *SAMM50*, no significant difference was observed between the ad_lncSAMM50 group and the ad_EGFP group (Figure 5E).

## Discussion

This study characterizes the lncRNA expression profiles of buffalo adipose and muscle tissues based on RNA sequencing analysis and evaluates the effects of lncSAMM50 on the adipogenesis of buffalo adipocytes. This study demonstrates that (1) the expression profiles of lncRNAs in buffalo adipose and muscle are significantly different with each other; (2) lncSAMM50 is a nuclear-location non-coding RNA; (3) *SAMM50* inhibits adipogenic differentiation in 3T3-L1 cells; and (4) lncSAMM50 promotes adipogenic differentiation by slightly upregulating *PPARG*, *C/EBP*α, and *LPL* in buffalo adipocytes, but with no effect on its host gene *SAMM50*.

Each of the activities in living organisms is precisely mediated by a genome. Generally, the gene is expressed in a time- and stage-specific manner and is regulated by multiple factors. With the development of RNA sequencing, the lncRNA expression profile has been characterized in multiple tissues in livestock animals (Huang et al., 2019; Li et al., 2020; Song et al., 2020). In the present study, the comparison of the lncRNA expression profiles of adipose and muscle tissues identified 241 DE lncRNAs (Supplementary Table 3). The quality of the differential expression analysis was further identified by qRT-PCR. These results indicated a significant difference in the biological function between adipose and muscle tissues in buffalo. Among the DE lncRNAs, four with high expression are significantly upregulated in adipose tissue (Table 1). The *NDUFC2*-AS lncRNA (TCONS_00096612) has been demonstrated to promote the adipogenesis in buffalo adipocytes (Huang et al., 2019). FABP4 is a significant protein in fatty acid transportation (Boord et al., 2002) and adipocyte differentiation (Garin-Shkolnik et al., 2014). SAMM50 is a mitochondrial membrane protein and is associated with energy metabolism in mammals (Liu et al., 2016). Considering the function of host genes and the lowest p value (Table 1), we focused on a lncRNA transcribed from the antisense strand of *SAMM50*, lncSAMM50. Interestingly, lncSAMM50 is mainly expressed in adipose tissue (Figure 3C) and is upregulated during the adipogenic differentiation of buffalo adipocytes (Figure 3E). These results indicated a vital role of lncSAMM50 in fat deposition of buffalo (Li et al., 2016; Huang et al., 2019).

The existing data suggest that lncRNA can play a role by regulating the expression of a host gene (Guo et al., 2019; Song et al., 2020), meaning that the function of a lncRNA is associated with its host gene. SAMM50 is the core component of the sorting and assembly machinery and plays a critical role in regulating mitochondrial dynamics and mitophagy (Liu et al., 2016; Jian et al., 2018), indicating a significant role of SAMM50 in energy metabolism. In the present study, we found that SAMM50 is widely expressed across different tissues in buffalo, especially in tissues with high level in energy metabolism such as the heart, liver, muscle, and adipose (Figure 3D). These results are consistent with its vital role in mitochondria (Liu et al., 2016; Jian et al., 2018). However, the effect of SAMM50 on adipogenic differentiation of adipocytes had not been revealed. By gain-of-function experiments, we demonstrated that SAMM50 inhibits the adipogenic differentiation of 3T3-L1 adipocytes (Figures 4D–F). These results further indicate that lncSAMM50 may affect the fat deposition by regulating the expression of its host gene SAMM50.

To confirm the effect of lncSAMM50 on fat deposition, an overexpression of lncSAMM50 in buffalo adipocytes was performed by an efficient adenovirus system. As expected, lncSAMM50 significantly enhances the lipid accumulation in buffalo adipocytes (Figures 5C–E). Meanwhile, eight lipid metabolism-associated genes, including two adipogenesis markers *PPARG* and *C/EBP*α, two fatty acid uptake markers *FAT/CD36* and fatty acid-binding protein 4 (FABP4), two lipogenesis markers glycerol-3-phosphate acyltransferase 4 (*GPAT4*) and diacylglycerol *O*-acyltransferase 1 (*DGAT1*), and two lipolysis markers lipase E (*LIPE*) and *LPL*, were used to predict the potential regulatory mechanisms of lncSAMM50 in buffalo adipocytes. PPARG and C/EBPα are well known as the crucial determinants of adipogenesis in adipocytes (Lowe et al., 2011; Mota de Sá et al., 2017). With the significant increase of lncSAMM50, both *PPARG* and *C/EBP*α were slightly upregulated (Figures 5E,F). These results indicate that lncSAMM50 may not have a direct impact on the expression of *PPARG* and *C/EBP*α but promote the adipogenic differentiation of buffalo adipocytes. FABP4 is a member of the fatty acid-binding protein family which is responsible for the intracellular transport of fatty acids (Lappas, 2014). FAT/CD36 is a membrane protein expressed in adipose tissue and plays an important role in the transport of fatty acid into adipocytes (Bonen et al., 2007). LPL can be produced by adipocytes and transferred to the surface of adipocytes to hydrolyze triglycerides and liberate free fatty acids (Merkel et al., 2002; Yagyu et al., 2003). The fatty acid produced by LPL lipase can be transported into adipocytes, synthesized again, and stored in adipose tissue (Merkel et al., 2002). In the present study, though the expression of FABP4 was not stimulated and the expression of FAT/CD36 was slightly inhibited by lncSAMM50 (Figure 5G), the expression of *LPL* was slightly upregulated (Figure 5I) in the ad_lncSAMM50 group, indicating that lncSAMM50 may enhance the fatty acid transport into buffalo adipocytes. GPAT4 and DGAT1 are key markers for triglyceride synthesis (Lappas, 2014; Yan and Ajuwon, 2015). Regretfully, both GPAT4 and DGAT1 were not stimulated by the overexpression of lncSAMM50 (Figure 5H), indicating that lncSAMM50 has no effect on the expression of these two genes.

Existing evidence suggests that lncRNAs can repress or activate the hose gene in the *cis* method (Fatica and Bozzoni, 2014; Wang et al., 2016; Song et al., 2020). Sirt1 antisense (AS) lncRNA is transcribed from the AS strand of the Sirt1 gene. Sirt1 AS lncRNA promotes myoblast proliferation and inhibits differentiation by interacting with Sirt1 3′UTR to rescue Sirt1 transcriptional suppression by competing with miR-34a (Wang et al., 2016). Similarly, another lncRNA IGF2 AS promotes the proliferation and differentiation of bovine myoblasts by complementing the IGF2 intron and affecting the expression of IGF2 mRNA (Song et al., 2020). In the present study, the sequence of lncSAMM50 is reverse complementary to the upstream region, exon 1, and part of intron 1 of *SAMM50* (Figure 2A). Additionally, lncSAMM50 is a nuclear localization transcript (Figures 2D,E). Thus, the physical proximity of lncSAMM50 and *SAMM50* inspired us to investigate a relationship in regulation between them. Unfortunately, overexpression of lncSAMM50 does not affect the expression of *SAMM50* in buffalo adipocytes (Figure 5E). Previously, we also identified a similar lncRNA, *NDUFC2*-AS lncRNA, which promotes the adipogenic differentiation by upregulating adipogenesis relative genes but with no obvious effect on the host gene as well (Huang et al., 2019). Thus, the precise regulatory mechanism of lncSAMM50 promoting the adipogenesis of buffalo adipocytes still needs further investigation.

Meanwhile, limitations still exist in this study. Firstly, the sample size (*n* = 3) and the gender (male only) for RNA sequencing seem to be limited. A higher sample size and use of both male and female animals will harvest a more accurate expression profile of lncRNAs. Secondly, identification of the effect of SAMM50 activity in buffalo adipocytes will contribute to a clearer relationship between SAMM50 and lncSAMM50. However, the effect of SAMM50 on lipid accumulation in adipocytes was only evaluated in the 3T3-L1 cell line but not in buffalo adipocytes. This is because the transfection by a simple liposome method is practicable in 3T3-L1 cells but not in buffalo adipocytes. Moreover, overexpression must be performed through the more complex and time-consuming virus system in buffalo adipocytes.

In conclusion, the present study provides a valuable genomic resource for identification of functional lncRNAs in buffalo and reveals the important role of lncSAMM50 in lipid accumulation of buffalo adipocytes. These data further perfects the molecular theory on buffalo fat deposition, which will instruct the buffalo breeding by genetic engineering or genome editing.

## Data Availability Statement

The RNA sequencing data were deposited in the GEO profiles of NCBI. The accession number of three adipose tissues is GSE112744 and that of three muscle tissues is GSE139102.

## Ethics Statement

The animal study was reviewed and approved by Institutional Animal Care and Use Committee (IACUC) of Xinyang Normal University.

## Author Contributions

JH, QL, and DS designed the experiment. XF, YW, and RZ collected the samples. XF, YW, RZ, DG, and JL performed the experiments. JH and XF analyzed the data and wrote the manuscript. All authors have read and approved the manuscript.

## Conflict of Interest

The authors declare that the research was conducted in the absence of any commercial or financial relationships that could be construed as a potential conflict of interest.

## Supplementary Material

Supplementary Table 1 Details of primers used for qRT-PCR detection.

Supplementary Table 2 Total lncRNAs identified in the adipose and muscle tissues of buffalo.

Supplementary Table 3 The lncRNAs showed differential expression between adipose and muscle tissues in buffalo.

Supplementary Table 4 Full length of lncSAMM50.
