# Supplementary material for: lncSAMM50 Enhances Adipogenic Differentiation of Buffalo Adipocytes With No Effect on Its Host Gene
Source: Front Genet. 2021 Mar 26;12:626158. doi: 10.3389/fgene.2021.626158 (PMC8033173; doi:10.3389/fgene.2021.626158)
Supplement: Supplementary Table 4 — Full length of lncSAMM50. [file Table_4.DOCX]

Table S4 Full length of lncSAMM50.

| Name | Length | Sequence |
| --- | --- | --- |
| lncSAMM50 | 3169 nt | CTAGCCTTCGGGCCCCACCGCCCTCTGTTAACCCGGGCAGCCCCGGGCGCAGCCGCGAGAGCGTGTCCCCGCGGCGCCCGCCCCGAGGTCACGCTGCAAGCGCGGGTCGCTGGGCCTCTTACCCGGGCGTGCACCGTCCCCATGGCTCCCTCTCTGGCCGCCGCCTCCCAGAGCAGTCCCGGCCGGACGAAGGCTCGGCGGGTGAGGACAGAGGGCGGGGCTCCGGTGACCGAACTCCGGGTTAAGGCAACACCCACAAGCCCCTAGACGCGGCGGACTGGGGGCGGCCATGATGTCCCGCCTTCGTCCCGCCCCTTGGCTCTCGGTGCTTCCGCTCATTGGCTCATAGGCGTCCGTGACCCGGATGTACACGCGGACGCCCGGAAGCCCTGAGACCCAGAGACGGCCCCAGCGCCGCGCGTTCGTTAGCGCTCAGAGGAGCTGGCCGCGAGTGGACGGCGGGTCTCCGTGGGTATCTAGTGGGGGTGGGCGCCCCGAGTTACCCCTGGGGGACAGTACGCCAGTTGGTGAGCGTGTCGGGCCTCGTGTCTTTCCCTTAAACCACGTGTTTAGTTCAGTTCAGCCGCTCAGTCGTGTCAGACTCTTTGCGACCCCATGGACTGCAGCACGCCAGGCCTCCCTGTGCATCGCCACCTCCTCGGAGTTTGCTCAAATTCGTGTCCATGTCCTTGGCTCCGCGGTGCCTCTCTTGTCCCCGCACTCTGGCGAAGCGCCGCGGCTCTCCACGCCTGGCTGCGGCTGTGGCCTCCCCAGCCCCTGCCTGCGGAGCCTCACAAATCCGGATTGGCGCCCTGCACCTCTCCCGCTGGCCACATGGACCCTTCAGGTCTCTGAAGCTCCCAGGGCTCAGGCTCCTCGTTGACAGAATGAAGGTGACGTCGTACCTGCTTAGCAGGGATGAATTACAAGGCTTTTGCACGAGAGTATGGCGAGTGAGCAGCTAGTCAATGGCCCCTATTCTTTCAACTGCAGTAAATGTCAAGAGTGTTAACCGGGGGTTGACGTGGGGACTATTCACATTGAAAAATAAGACAGCTCTGGTGCTACTGAGCCTAAGCTCGCCCTGATCCTGGTAGGATAGGCAGTGAATCTGGGGAGAGGAGGAAATGAGGCCAGGAGTCTGACTTTATTTGGAAAGCTGGCTGACTGAGAAGAGAGCAGACTGATGTCTCAAAGTAACCCATCTTGTAGGGGTCTGGATGCCAGGTTCTTTTATGGATCTGAGATGGGGGAGGGGCGAGGAGACAAAGTAAAATAAAGGCCTTTAATCTTGGAAATATTCTTTTAGAATGCCAAGTCTCAGGCAGAGAGATATGTTAACTTCTTTCTTCCTGCCTTCTCCAGGTGGACAGGGTTATGCACAAAGGCACTTTAGCTTAACAGGCAGAGGGGCAGAATTCTCTGAGGCAGGGCATTATGTATGATTGTAATAACGAAAGCAACAGAAAGCAAGTGGAACAGTTTCAAGGTGGAGTCAGAATTGACTTCTCCCTGCAACGTGGGGCTTTATTTTGGTGGTGGAAACAGCTTCCTGTCTCAAATGACAGCATGTAATAAGTAATAGCACATGGTGATAAATGCAGTGAGAGAAACAAGAGAACGGTTTTCATTATCATGTGGTCTCACGTTTCCACCCCGTCCCAGAGACCCTGACACCCTTTCCCATTTAAGTAGCAGTACCTTAATTCTCTTGCCTTCCTGGGGTAAATATTAAGTCCATATTCCCCTTCTTGCCTGGGAAGAGACAAGACATTAGCTCCTCCCAGGCGCCCCTTCCCACACCGCATTCCTTGCCAGTTGACCTAGTCATCTGTTTGAGCTGGACTGGTGCCTGGAGGCGGAGCCCAGTCCTTCGCTCCATGAGCCCAGAGGCTGCATGGTGCCTGGCGTGGTGCAGGGACTCAGTTAATATTTCCCGAATTACTGCTCTCCCACCGTAGGCTACAGCAGTCAGAAAGACACTGCTTATATTAAAAATGCACATTCCTGGGCCTCAGCCCTGATCTACTGAACCAACCCCCGGAAAGTAGAACCTGGAGATATGAGTTTTTTAAAATGGGCTATATTATTCTTTTAGCCATTATGTTTAAGAACCCCACAGTAAAGAATCTGCCTGCGGTGCGGGTTCCGTCCCTGGGTGGGGAAGATCCCCTGGAGAAGGAAATGGCAACCCACTGCAGTATTCTTGCCTGGGAAATGCCATGGACAGAGGAGACTGGTGGCCTACAGTCCATGGGGTTGTAAAAGAGTCAGACACGACTTAGTGATTAAACAGCAACATTCTAAAAAGCTTTAGGTCTGTGTCTGACTTTATCACTCATTTCTAAAGTCTCTGAAGACTTGGGCTACTCACGTGCTTATAAAGGAACTTGGCCAGAATTGAGTGCAGAGGTGCTCAGCAAGGGGGAGGAGGAAGCAGAGGCAGCGGCAGCAACAGAGCATCTGCAAGCGGGGCAGGGCTGTGACAGAGGTCTGGCCCAGAGGCTTCTGGGTCTGGGCATACATTTGAAGGACAGCGAGATGATGGAAGAACTGGGCTGTAAGTTAGCAGTTGACCCCTGGCAAGTCACACCCTCTTTAAGGGTTACTTTTCTCCACTGTAAAATAGAGATGAGCTTGCTAAGGACCAAAAACAGAAGGTTTTTGTTTTACATGTTTTATATTTCACACTGGGAAGTAACAGGTCCTGGGCTTTTGTACTCGTTTAGGCCGTTTTGGATCACCCCTTGGCAGGAGATCAAAACCAGATTCGCTCCCCATTAATGGTTACGAGAGCTGGCTTTATGGGCAGACCCGCTGGTTGGGTTTCAGCCATGATTCTGTCACTCTTAAGCTGTGTGGACTTAGGCAGGGCCTGTACCTCTCTGGGTCAGTTTCCCCTTTGGTGAAGTGAGGGTGGTGATGGTATCTACCTCATAAGGTTAGTAAATAATGTAAGGGTTTGCCTGGCCTAGAGAGAAAGGGCAGGTGCACACGCACGCATGCACAGACACACAGCCTGCAACCAGGCCTAGAGAGAGCACGTGTGCACACGTGCACACAGACACACACACAGACTGCAACCAGGCCTAGAGGGAGAGAGCGCATGTGCACACACACACACACACACACACACACACACACAGCTGCAACCAGGCCTAGAGGGAGAGAGCACGTGTGCACACGCACACACACA |
